# Retraction: Phospho-Aspirin-2 (MDC-22) Inhibits Estrogen Receptor Positive Breast Cancer Growth Both *In Vitro* and *In Vivo* by a Redox-Dependent Effect

**DOI:** 10.1371/journal.pone.0341199

**Published:** 2026-01-16

**Authors:** The PLOS One Editors

Following the publication of this article [1] concerns were raised about results presented in Figs 3-5, and 7. Specifically,

- The following panels appear to overlap:
  - β-actin data in Fig 3A (T-47D cells), Fig 4A (lower panel), and Fig 4B (bottom panel)
  - β-actin data in Fig 3B and Fig 4A (upper panel)
  - β-actin data in Fig 3D and Fig 4B (middle panel)
  - Fig 4A upper panel p53 and Fig 7A β-actin
  - Fig 5A MCF-7 panels for Sam 68 (Cyto lanes) and β-actin (Mito lanes)
- The labelling for multiple panels in Figs 3 and 4 is incomplete, such that it is unclear which results were obtained from MCF7 cells and which results were obtained from T-47D cells.

The corresponding author stated that the original data underlying the published results are no longer available.

In light of the unresolved concerns, which call into question the reliability and integrity of the published results, the *PLOS One* Editors retract this article.

LH and BR did not agree with the retraction and stand by the article’s findings. CCW and KWC either did not respond directly or could not be reached.
